# Skill Memory Escaping from Distraction by Sleep—Evidence from Dual-Task Performance

**DOI:** 10.1371/journal.pone.0050983

**Published:** 2012-12-04

**Authors:** Denis Ertelt, Karsten Witt, Kathrin Reetz, Wolfgang Frank, Klaus Junghanns, Jutta Backhaus, Vera Tadic, Antonello Pellicano, Jan Born, Ferdinand Binkofski

**Affiliations:** 1 Department of Neurology, University of Lübeck, Lübeck, Germany; 2 Department of Neurology, University of Kiel, Kiel, Germany; 3 Department of Neurology, Rheinisch-Westfälische Technische Hochschule Aachen University, Aachen, Germany; 4 Institute of Neuroscience and Medicine, Research Center Jülich, Jülich, Germany; 5 JARA BRAIN – Translational Brain Medicine, Rheinisch-Westfälische Technische Hochschule Aachen University, Aachen, Germany; 6 Neurological Practice, Bad Schwartau, Germany; 7 Department of Psychiatry, University of Lübeck, Lübeck, Germany; 8 Department of Neuroendocrinology, University of Lübeck, Lübeck, Germany; 9 Division for Clinical and Cognitive Neurosciences, Department of Neurology Medical Faculty, RWTH Aachen University, Aachen, Germany; Harvard Medical School, United States of America

## Abstract

**Background:**

Sleep facilitates off-line consolidation of memories, as shown for learning of motor skills in the absence of concomitant distractors. We often perform complex tasks focusing our attention mostly on one single part of them. However, we are equally able to skillfully perform other concurrent tasks. One may even improve performance on disregarded parts of complex tasks, which were learned implicitly. In the present study we investigated the role of sleep in the off-line consolidation of procedural skills when attention is diverted from the procedural task because of interference from a concurrent task.

**Methodology/Principal Findings:**

We used a dual-task paradigm containing (i) procedural serial reaction time task (SRTT), which was labeled as subordinate and unimportant and (ii) declarative word-pair association task (WPAT), performed concomitantly. The WPAT served as a masked distractor to SRTT and was strongly reinforced by the instructions. One experimental and three control groups were tested. The experimental group was re-tested after two nights of sleep (*sleep group*, *SG*). The first control group had sleep deprivation on the first post-learning night (*nighttime-awake group*, *NA*), the second control group was tested in the morning and then re-tested after 12-hours (*daytime-awake group*, *DA*); the third one had the same assignments as *DA* but with a subsequent, instead of a concomitant, WPAT (*daytime-awake-subsequent-WPAT* group, *DAs*). We found SRTT performance gains in *SG* but not in *NA* and *DA groups*. Furthermore, *SG* reached similar learning gains in SRTT as the *DAs* group, which gained in SRTT performance because of post-training interference from the declarative task.

**Conclusions/Significance:**

The results demonstrate that sleep allows off-line consolidation, which is resistant to deteriorating effects of a reinforced distractor on the implicit procedural learning and allowing for gains which are consistent with those produced when inhibited declarative memories of SRTT do not compete with procedural ones.

## Introduction

Sleep following learning of procedural tasks without distraction by declarative tasks [1]–[7], and learning of declarative tasks [8]–[10] leads to an improved performance at a delayed retrieval testing [9], [11]–[14]. Skill performance at retrieval after post-training sleep can be even better than immediately after training before sleep, a mechanism which has been ascribed to an “off-line learning” process that goes on after training has terminated and which is enhanced by sleep [15]–[17].

In daily life, learning a procedural task is often distracted by other tasks. Nevertheless we are able to learn and improve performance. This phenomenon has been addressed by previous studies adopting dual task paradigms where, e.g., a simple tone counting task was used as a distractor while subjects performed a serial reaction time task (SRTT) [18], [19]. In the SRTT a visual cue must be matched to a certain motor response as fast as possible. Unnoticed by the participant the sequence of cue positions follows a fixed serial pattern leading to decreased reaction times in the fixed sequence as compared to reaction times to random sequences of cue positions. This task has been used widely in isolated form and as part of a dual-task setting to assess mechanisms of implicit skill acquisition [19]–[24].

Learning the SRTT in a dual task setting, i.e., while distracted by performing a tone counting task in parallel, revealed basically two effects: first, a greatly diminished probability of the subject to become aware of the fixed sequence and second, the distractor task diminishes gains of response times on the fixed sequence [25]. Tasks like the SRTT comprise explicit declarative aspects in addition to their implicit procedural aspect. Both may interact and compete during task processing, with this competition continuing off-line after training. Thus, Brown and Robertson [26] showed a performance gain in SRTT skill at delayed retesting, if subjects had to perform a declarative word-pair association task (WPAT) immediately after the first training of the SRTT sequence. They argued that procedural consolidation can be disrupted by declarative learning and that the reverse is also true, with declarative consolidation being disrupted by procedural learning. But, they also propose that sleep provides optimum conditions for off-line skill consolidation because during sleep procedural and declarative memory systems become disengaged from any competitive interaction [26].

Here, we investigated the effects of word-pair learning concurring with SRTT training, i.e., the effects of a dual task setting supposed to be more comparable with everyday life. Our subjects performed the SRTT as an incidental learning task, while a concomitantly performed word-pair association task was declared as the important learning task and was additionally reinforced with a monetary reward. One group of subjects slept the night after training (*sleep group, SG*), whereas another group remained awake on this first night (*nighttime-awake group, NA*). Retrieval was tested after a second night during which both groups slept. A third group was tested in the morning and retested in the evening after a 12-hour retention period without sleep (*daytime-awake group, DA*). A fourth group was like the *DA*, but similar to Brown and Robertson [26] subjects performed the SRTT and the WPAT one after another (*daytime-awake-subsequent-WPAT group, DAs*). This allowed to assess the influence of procedural encoding during the SRTT and the day-time consolidation in a concurrent, dual-task setting compared to a subsequent tasks setting.

On the one hand, based on Brown and Robertson’s [26] findings, word-pair learning during SRTT training, like word-pair learning shortly after SRTT training, can be expected to allow for substantial off-line gains in SRTT performance even when subjects remain awake after training, due to an inhibitory effect of word-pair learning on declarative aspects of SRTT competing with procedural SRTT memories. On the other hand, however, when introduced during SRTT training, word-pair learning may function as additional declarative input that impairs the procedural aspects of SRTT, thus generating costs in SRTT performance (dual-task interference costs). In this case, a gain in skill performance would be expected for the post-training sleep condition (sleep group) only, as compared to conditions without post-training sleep (nighttime-awake, daytime-awake and daytime-awake-subsequent-WPAT groups). We could show that this was the case. Indeed, we demonstrated the positive effect of sleep on the consolidation of procedural learning, even when a directly reinforced distractor was used while the procedural learning task was interpreted as unimportant by the subjects.

## Results

### SRTT Performance Across All Time-points of Assessments

**Figure 1:**
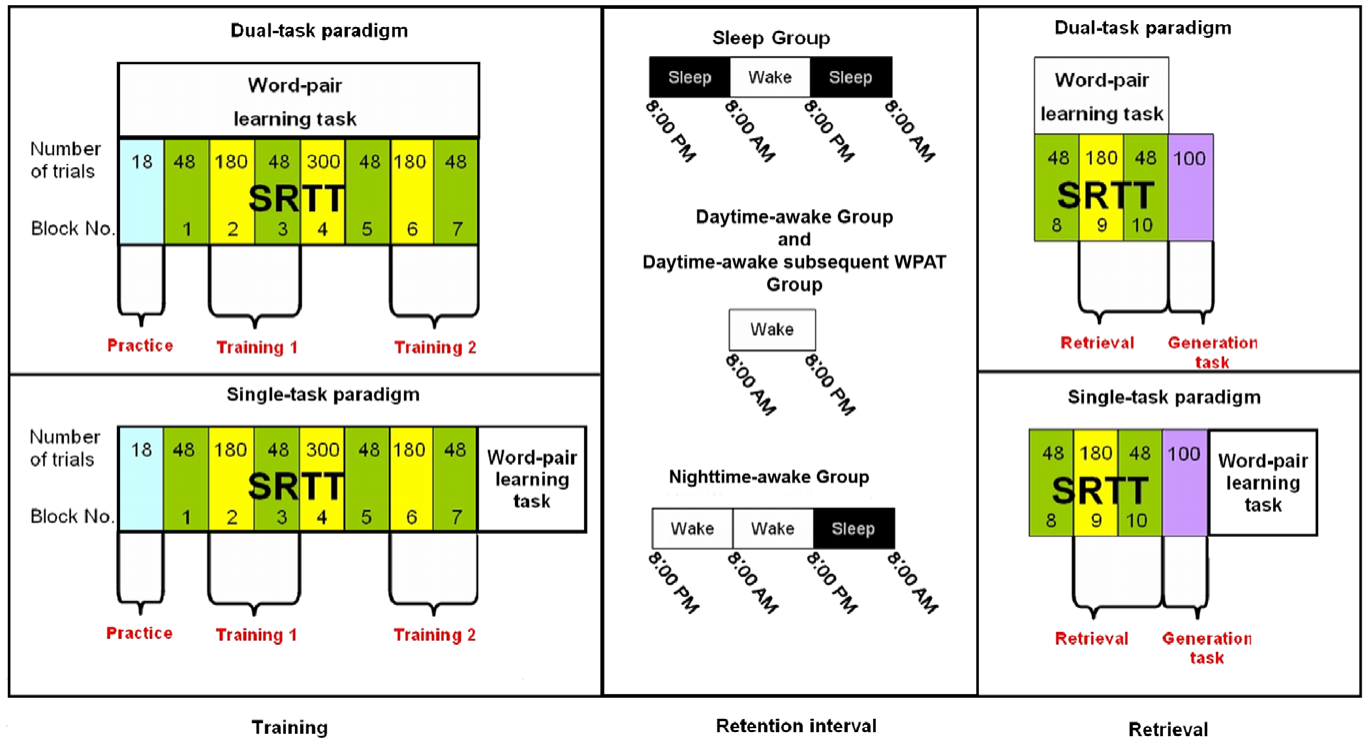
Experimental procedure. The experiment consisted of a *Training* followed by a *Retrieval* session. Sleep group (*SG*), nighttime-awake group (*NA*) and daytime-awake group (*DA*) performed a dual task (concomitant SRTT and WPAT; upper part). The daytime-awake-subsequent-WPAT group (*DAs*) performed a single task (SRTT alone then followed by the WPAT; lower part). *Training session (left part)*. SRTT was arranged in blocks (in yellow), for which the trial sequence was *fixed*, followed by blocks (in green) with *random* sequences of trials. Numbers in the bottom mark block’s position in the general sequence whereas, numbers on the top indicate the number of trials administered within each block. “Practice” (light blue) indicates the initial practice block, which was not included in the analyses. The WPAT (distractor task) was continuously performed throughout the entire SRTT session. The first and the last fixed sequence blocks (i.e.: block 2 and block 6), followed by the random sequence block 3 and 7, respectively were considered to investigate changes in performance across *Training*. For clarity, block 2 and block 3 were labeled as “Training 1” whereas block 6 and block 7 were labeled as “Training 2”. The difference between mean RTs to random and sequential blocks was the dependent variable being analyzed. *Retention interval between training and retrieval sessions (middle part)*. The sleep group slept the two nights after *Training* before doing *Retrieval* in the morning. The nighttime-awake group, instead, stayed awake throughout the first night after *Training* and slept the second night. The daytime-awake group trained in the morning and was retested in the evening of the same day during which they stayed awake. *Retrieval session (right part)*. Block 9 (fixed trial sequence) and block 10 (random trial sequence) were labeled as *Retrieval*. The same random minus fixed block difference as in the training session was taken to investigate the RT performance. The task was immediately followed by one bock (in purple) in which a generation task was administered.

To analyze the SRTT performance across the Training and the Retrieval sessions, “delta” reaction times (RTs) were calculated. For the Training session, mean RTs to the first and the last fixed sequence blocks (block 2 and block 6, respectively) were subtracted from the mean RTs to the subsequent, random sequence blocks (block 3 and block 7). Thus, *Delta Training 1* and *Delta Training 2* measures were obtained, respectively (Figure 1, left part). For the Retrieval session mean RTs to the fixed sequence block 9 were subtracted from the mean RTs to the random sequence block 10. The *Delta Retrieval* measure was then obtained (Figure 1, right part).

An analysis of variances (ANOVA) with *Group* (*sleep-SG* vs. *nighttime-awake-NA* vs. daytime-awake-DA vs. *daytime-awake-subsequent-WPAT*-DAs) and *Time-points* (*Delta Training 1* vs. *Delta Training 2* vs. *Delta Retrieval*) as between- and within-subjects factor was performed.

A significant *Group* x *Time-points* interaction was observed F_6, 66_ = 3.03, p = 0.011, partial-η^2^ = 0.2 which was further investigated by means of two separate ANOVAs to assess SRTT performance during training and from training to retrieval sessions.

### SRTT Performance During Training

An ANOVA with *Group* (*SG* vs. *NA* vs. *DA* vs. *DAs*) and *Training* (*Delta Training 1* vs. *Delta Training 2*) as between- and within-subjects factors was performed.

The main effect of group was significant F_3, 33_ = 5.19, p = 0.005, partial-η^2^ = 0.3. Performance of the *DAs* group resulted distinctively better than the other three groups (see figure 1, left side), although post-hoc Scheffé test showed significantly greater delta RTs for *DAs* respect to *NA* p = 0.029 and *SG* p = 0.008 but not to *DA* p = .132.

This result points to a possible circadian effect on group performances, so that the groups that trained in the evening would have performed worse than those who trained in the morning. Although on the one hand, we cannot exclude some participation of this factor to the main results; on the other hand, we consider it as marginal compared to the substantial effect of WPAT on SRTT.

Crucially, all four groups showed improvement in SRTT performance during the training session, as indicated by the significant main effect of *Training* F_1, 33_ = 36.80, p<0.001, partial-η^2^ = 0.5 and the non-significant *Group* x *Training* interaction F_3, 33_ = 1.22, p = 0.32, partial-η^2^ = 0.1 (see table 1, 2). Indeed delta RTs had similar increase from the first (Training 1) to the very last training sequence (Training 2) for all groups.

### Off-line Consolidation Effects

For the analysis of off-line gains in RTs an ANOVA with *Group* (*SG* vs. *NA* vs. *DA* vs. *DAs*) and *Retrieval* (*Delta Training 2* vs. *Delta Retrieval*) as between- and within-subjects factors was run. This analysis revealed a significant main effect of *Group* F_3, 33_ = 7.64; p = 0.001 partial-η^2^ = 0.4. Post-hoc Scheffé tests revealed greater delta RTs for the *DAs* group compared to *DA* p = 0.001 and *NA* p = 0.022 groups.

**Figure 2:**
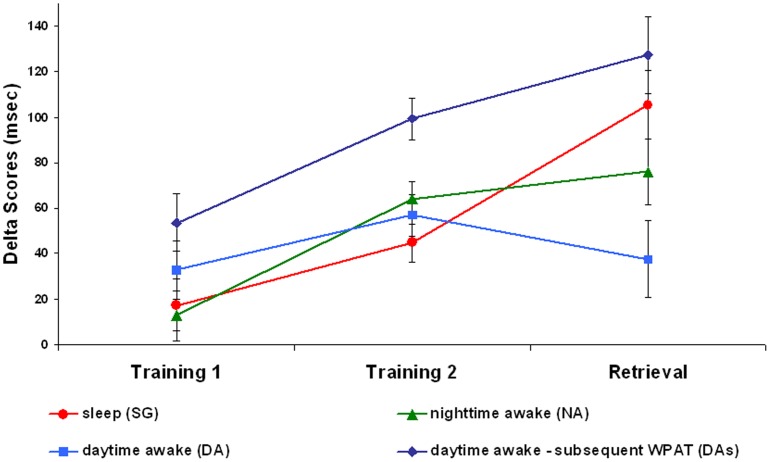
Graph of SRTT performance gains across the three time-points of assessment. Delta means improvement in SRTT performances (in msec) for the four groups during the Training session (left graph) and in the Retrieval session compared to Training 2 (right graph).

More interesting, significant *Retrieval* main effect F_1, 33_ = 5.31; p = 0.028 partial-η^2^ = 0.1 and *Group* x *Retrieval* interaction F_3, 33_ = 3.54; p = 0.025 partial-η^2^ = 0.2 were observed (see Table 1, 2). An overall improvement in SRTT performance was produced in the Retrieval compared to the Training 2 point of assessment. However, post-hoc paired-sample t-tests specified that the increase in performance gain took place in the *DAs* group T_7_ = 3.53, p = 0.010 and, crucially, in the *SG* group T_9_ = 2.41, p = 0.039 but not in the NA T_10_ = 1.04, p = 0.324 and *DA* T_7_ = 1.08, p = 0.314 groups (Figure 2). Furthermore, vertical comparisons between *SG* and *DAs* group revealed that as a product of both dual-task interference costs in the *SG* and gains in procedural aspects of SRTT in the *DAs*, a worse performance was observed in the *SG* compared to the *DAs* group for the SRTT training session T_16_ = 5.06, p<0.001. However at the retrieval stage, performance of the *SG* increased to a level similar to the one reached by the *DAs* group, so that no significant difference was observed between the two groups T_16_ = 0.84, p<0.415. Thus, consolidation processes active during sleep were able to not only overcome the initial lack of procedural skills developed in the training task, but also raise the level of performance to the benefited one showed by the *Das* group.

**Table 1:** Descriptive statistics and post-hoc analyses of SRTT performance across Training 1 and Training 2 and across Training 2 and Retrieval, for all groups.

	Average delta reaction time improvement[mean ± SEM] from Training 1 to Training 2;t-test values	Off-line gains in reaction times[mean ± SEM] from Training 2 to Retrieval
**Sleep group**	27.5±10.0 msec;t = −2.74, p<0.05, d = −1.22	60.5±25.1 msec,t = 2.41, p<0.05, d = −0.88
**Nighttime-awake**	51.1±10.9 msec;t = −4.69, p = 0.001, d = −1.19	12.0±11.6 msec,t = −1.04, p = 0.32, d = −1.63
**Daytime-awake**	24.2±9.3 msec;t = −2.59, p<0.05, d = −0.7	−19.4±17.8 msec,t = 1.08, p = 0.31, d = −0.93
**Daytime-awake-** **subsequent-WPAT**	45.6±17.8 msec;t = −2.56, p<0.05, d = −0.9	28.1±7.9 msec,t = −3.53, p = 0.01, d = −1.33
**ANOVA**	F_3,33_ = 1.22, p = 0.32, partial-η^2^ = 0.1	F_3,33_ = 3.54, p<0.05, partial-η^2^ = 0.24

Beside mean and standard error of mean (SEM), the results of paired-sample *t*-tests are presented (t = t-value, p = significance value, d = Cohen’s d). In the last row, the *Group* x *Training* and *Group* x *Retrieval* interactions from the respective separate ANOVAs are reported.

**Table 2:** Mean reaction times in msec (+/− SEM) for the three time-points of assessment of SRTT performance: Training 1, Training 2 and Retrieval.

	Mean (± SEM) msec in SRTT performance								
	Training 1			Training 2			Retrieval		
**Block properties**	**Fixed**	**Random**	**Delta** **(Random-** **Fixed)**	**Fixed**	**Random**	**Delta** **(Random-** **Fixed)**	**Fixed**	**Random**	**Delta** **(Random -Fixed)**
**Block No.:**	2	3	3–2	6	7	7–6	9	10	10–9
**Sleep group**	459.2 (14.9)	476.5 (14.9)	17.3(8.4)	411.2 (12.7)	456.1 (12.9)	44.8(5.5)	352.9 (14.23)	458.2(21.9)	105.3 (22.9)
**Nighttime-awake group**	496.7 (16.8)	511.2 (10.9)	14.5(14.2)	433.9 (10.4)	497.8 (13.9)	63.9(8.2)	386.3 (10.3)	462.2(11.8)	75.9(9.7)
**Daytime- awake group**	558.2 (25.8)	591.0 (21.8)	32.8(13.1)	481.7 (25.7)	538.7 (27.9)	57.0(11.4)	446.6 (20.7)	484.2(14.2)	37.6 (16.9)
**Daytime-awake-** **subsequent-WPAT group**	471.0(36.8)	524.7(33.6)	53.7(9.8)	462.7(30.9)	562.0(31.1)	99.3(10.0)	390.5(22.0)	517.9(24.7)	127.4(6.9)

For each group, block number and property are reported. “delta” indicates the differences between mean reaction times in the fixed and in the following random sequence blocks.

### Sleep Duration, Sleep Quality and Alertness

All groups were reporting a comparable duration of sleep prior to the experiment, and a comparable high global Pittsburgh Sleep Quality Index (PSQI) score (p>0.06 for all comparisons), indicating comparable sleep quality before the participation in the experiment. Prior to the start of the training session a standard adjective checklist was administrated assessing subjective activation, concentration and mood (“Eigenschaftswörterliste” (EWL-K) scores). The results revealed no difference between the groups, with high scores for activity and low scores for inactivity and fatigue (p>0.1 for all comparisons).

The sleep in the first night after the training session was assessed for the sleep group only, showing a duration of 6.7±0.3 h (SEM) and a good sleep quality (PSQI score mean 2.2±0.2 SEM). The sleep in the night prior to the retrieval session (i.e., the second experimental night with nightly sleeping) was not significantly different between the *SG* and the *NA* group according to duration and quality.

The standard adjective check list showed a high activity score and low fatigue score with no significant difference between the four groups, however a significant difference was found for the inactivity scores. Post-hoc pair-wise comparisons revealed a significant difference between *NA* and the other dual-task groups (*NA* vs. *DA*: p<0.05; *NA* vs. *SG*: p = 0.01; Scheffé contrasts). However, the remaining groups did not differ, and furthermore no difference was found between *NA* and *DAs*.

There was no significant correlation between sleep duration and off-line gains either for *NA* or *SG* (p>0.1). The same was true for sleep quality and off-line gains (p>0.3), as well as for the scores activity, inactivity, and fatigue in the adjective list (p>0.1).

### Generation Task

When a participant was able to choose 50 to 60% correct responses in a generation task, then relevant explicit knowledge of the hidden rule was assumed [19]. Using this as exclusion criteria for participation, no subject included into the final analysis gained significant awareness about the existence of the fixed SRTT sequence (percentage of correct reactions: *DA* group: 39.1%, *SG* group 40.5%, *NA* group: 38.8%, *DAs* group: 53%). Furthermore, the four groups differ systematically regarding this parameter (one-way ANOVA: F_3, 33_ = 3.37, p = 0.03, partial-η^2^ = 0.23). Subjects from the night groups (*SG* and *NA*) did not reach the 60% criterion of correct responses in a generation task and got insight of the hidden rule of the SRTT. However, subjects from the *DAs* group had more than a half of all responses being correctly chosen. The latter may be the effect of the absence of the distractor. A hint to this is given by the post-hoc Scheffé contrasts presenting a significant difference between *NA* and *DAs* (p = 0.03 [one-sided]), but not between the other groups.

### WPAT Performance During SRTT

As done for the procedural task, we compared the differences in word-pair association performance between the whole *Training* session (grouping Training 1 and Training 2) and the *Retrieval* session of the three groups performing the dual-task paradigm (i.e., *DAs* was not included in the analysis). As a result, we found no significant difference between groups (one-way ANOVA, F_2, 26_ = 1.16; p = 0.3; partial-η^2^ = 0.08). Indeed, the three dual-task groups performed similarly on the distractor task and most likely experienced an equal distractive effect. Additionally, these results indicate a similar ability to process the word-pair association during the first session.

Further analysis of the declarative task during the dual-task condition consisted in the comparison between the performances of both sessions. The analysis of variance showed a significant effect for *Session* (ANOVA, F_1, 25_ = 78.84, p<0.0001 [one-sided]), but not for *Group* (ANOVA, F_2, 25_ = 1.17, p = 0.16 [one-sided]) and no significant interaction (ANOVA, F_2, 25_ = 1.17, p = 0.3 [one-sided], therefore the differences between sessions showed a gain in performance for all groups from the first to the second session. The improvements were significant as shown by post-hoc paired sample t-tests on the group level. The absence of any difference in performance between the groups in the Retrieval session indicates a similar effect of off-line consolidation independent from the time interval between sessions (Table 3).

**Table 3:** Means and standard error of mean (SEM) percentages for correct word-pairs recalls during the SRTT processing, in training and retrieval sessions.

	Correct recall during SRTT in % (Training session)[mean ± SEM]	Correct recall during SRTT in %(Retrieval session)[mean ± SEM]	Gain in correct recall during SRTT from Training to Retrieval session in %[mean ± SEM]
**Sleep group**	55.5±5,4	72.5±9.4	17±9.6t = 1.78,p = 0.01
**Nighttime-awake group**	52.6±7,6	84.3±6.1	31.713±5.0t = 6.28,p>0.001
**Daytime-awake group**	41.3±6.4	67.0±8.1	25.7±5.5t = 4.67,p = 0.002
**Daytime-awake-subsequent-WPAT group**	-	-	-
**ANOVA**	F_2, 26_ = 1.12,p = 0.34partial-η^2^ = 0.08	F_2, 26_ = 1.26,p = 0.30partial-η^2^ = 0.09	F_2, 26_ = 1.16,p = 0.33partial-η^2^ = 0.08
**Post-hoc Scheffé test**	Not significant	Not significant	Not significant

Differences between both sessions are also shown.

Lastly, we compared the WPAT performance between groups including the *DAs* group. Here we found a significant difference only between *DA* and *DAs* for the correct answered recalls in the Training session (F_3, 33_ = 4.0, p<0.05, partial-η^2^ = 0.27; Scheffé: mean difference = 32.6% correct recalls, p<0.05); here the latter group had a better recall performance. No significant differences were found in the correct responses during the second experimental session or regarding differences between both sessions’ correct answers between all four groups.

## Discussion

In this study, we investigated the effect of sleep on the consolidation of implicit procedural information when a reinforced strong distraction is provided by a concomitant declarative task. Our results demonstrated that one night of sleep after the training session is sufficient to improve the procedural motor performance even when the concomitant distraction source is also provided in the retrieval session. By contrast, sleep deprivation in the first post-learning night or one daytime passed without sleep after learning prevented the emergence of off-line gains in SRTT performance at delayed retesting. Based on these results, we conclude that only off-line consolidation during sleep is able to reinforce implicit procedural knowledge learned under strong distraction. Apparently, consolidation during sleep has the unique capacity to shield newly acquired procedural skills from impairing influences of distraction arising from a dual-task setting. This finding may explain why we can improve procedural skills beside this may not be intended.

Previous studies have shown that performance on an SRTT improve significantly over a retention interval throughout the day (8 AM to 8 PM), when the procedural abilities are acquired implicitly in a single task setting. [7], [27]. This gain of performance is typically less pronounced than after a period of sleep. Moreover, underlying off-line consolidation without sleep appears to be fragile and sensitive to interfering influences. Indeed, repetitive transcranial magnetic stimulation (rTMS) over the primary motor cortex is able to eliminate gains developing during alert consolidation [28], [29]. Furthermore, daytime off-line consolidation is sensitive to retroactive interference: learning of a second sequence in a finger sequence tapping task prevents an off-line consolidation gain of a first trained sequence [4].

The aim of our study was to further investigate the daytime off-line consolidation when a dual task setting employing a strong distractor is implemented. Our results first display a significant effect of distraction over the implicit motor learnings in a dual-task setting. This can be seen from comparison of the *daytime-awake (DA)* and the *daytime-awake-subsequent-WPAT (DAs)* groups. Indeed, performance improved from Training 2 to Retrieval condition when the SRTT was performed alone whereas, it showed no change if the WPAT was performed concurrently to SRTT. Similar results were obtained in the *nighttime-awake (NA)* group who showed no gains in motor learning despite they did not sleep in the first night after the training, and were allowed to sleep the second night, before being retested in the retrieval session. This result rules out the general effects of fatigue which could have determined weaker performance in the *DA* group. This adds further support to the strong distracting effects of the concurrent declarative task on the implicit motor learning (dual-task interference costs).

Crucially, the group who was allowed to sleep also in the first night after training (*SG*) revealed an off-line improvement under the same dual task conditions provided for *NA* and *DA* groups. Indeed, performance to the SRTT improved significantly from Training 2 to Retrieval. Interestingly, as a product of interference of the WPAT on the SRTT a worse performance was produced in Training 2 by the *sleep (SG)* compared to the *DAs* group. However at the Retrieval stage performance of the *SG* group improved to a level similar to the one reached by the *DAs* group, so that no significant difference was observed between the two groups. This result allows us to conclude that the consolidation of implicit motor learning produced by sleep is strongly reliable. Indeed, sleep is able to overcome the initial deficit of procedural skills caused by the strong interference suffered in the learning phase and to bring performance in the Retrieval phase, up to the same level as the *DAs* group. Noteworthy the *DAs* group itself was supposed to foster gains in SRTT performance from Training 2 to Retrieval as a product of post-training effects of WPAT [26]. This further highlights the reliability of the sleep consolidation effects. In conclusion, our outcomes extend the previous observations of distinct procedural gains in SRTT skills (arising over an awake-retention interval) to the condition in which the two tasks (here SRTT and WPAT) are performed in parallel, rather than in sequence.

Within our results, possible biases due to differences in circadian position of the experimental sessions can be ruled out. In our study, both the *SG* and the *NA* groups had the Training and the Retrieval sessions administered at the same times of the day, so that eventual circadian differences biasing performance between the sessions would have produced equal effects in the two groups. As a result, differences in performance at the Retrieval compared to the Training between the two groups can, most plausibly, be re-conducted to consolidation effects of sleep.

These findings are in line with the concept of a two-stage process of motor memory consolidation: The representation of a motor skill is encoded in a temporary store during training. At this stage the consolidation process is slow and the representation is labile and remains susceptible to interfering inputs [28], [29] that can result (i) from rTMS applied after training over the primary motor cortex (M1), (ii) from successive training on a similar motor task or, (iii) as in the present study, from training the task under dual-task paradigms with a strong distractor. Our results thus show that distraction during training represents an interfering influence that continues during post-training periods without sleep and prevents the development of skill gains in the awaked brain. A period without sleep (daytime-awake group) and a period of deprived sleep (nighttime awake group) did not lead to any significant gain in the distracted SRTT performances.

Our results point to the fact that sleep is a preposition for consolidation of a motor memory encoded in a dual task set-up. These results fit very well into the theoretical framework of memory consolidation proposed by Robertson [15] which is centered on the mechanisms of disengagement. According the author, memory systems operate independently during sleep, but interact during wakefulness. Sleep may functionally disconnect the declarative and procedural systems, allowing them to operate as independent memory systems. Alternatively, the same neural resources may support procedural and declarative processing during wakefulness, whereas distinct resources may support memory processing during sleep.

### The Role of Sleep in Off-line Consolidation

Sleep-dependent motor memory consolidation causes an improvement in the motor performance compared to post-training awake intervals (e.g.) [3]–[5], [14]. In a study by Walker et al. [4] two different finger tapping sequences learned successively diminished sleep–dependent performance gains for the first-learned sequence due to interference effects. On the other hand, Korman et al. [28] showed that a 90-min nap introduced after training a finger tapping sequence suffices to stabilize the newly acquired motor sequence representation, which thereafter is less susceptible to interference. The mechanism behind this sleep-dependent process of system consolidation (performance gains and stabilization) was explained by a redistribution of motor memories to different neural networks, a concept that is also supported by several functional magnetic resonance imaging (fMRI) studies [29]–[32]. After sleep, the brain activity related to the finger sequence tapping skill shifted from ipsilateral primary motor and premotor areas to contralateral primary motor and striatal areas [31], [32]. Also increased hippocampal activity has been observed after sleep-dependent consolidation of finger motor skill [32].

Our results tie in with this previous body of evidence in showing profound performance gains in SRTT skill despite the dual task performance only in the group of subjects who slept after training. We assume the improvement resulted from a re-distribution and transfer of the newly acquired motor representations to other networks representing related to long-term storage. As a consequence, the new representation of the motor skill shows diminished susceptibility to distraction at retesting [28]. The redistribution of memory representations is probably a consequence of reactivation of the newly established representations during sleep [33]. We suppose this reactivation disentangles and detaches the skill representation from the interfering influence of the distractor task present at encoding (see also) [34] because in our study, the selective sleep-dependent gain in SRTT skill emerged from training under dual task conditions.

Effects of sleep on consolidation of finger sequence motor skills have been much more frequently studied using the finger sequence tapping task than the SRTT employed in our study. For this reason much of the foregoing discussion integrated studies investigating finger sequence tapping. However, the SRTT containing a distinct perceptual-motor component that differs from classical finger sequence tapping where motor responses depend solely on explicit representation of the motor outputs, because subjects move their fingers in a remembered sequence without any external cue [3]–[5]. There are significant differences between the mechanisms of learning and consolidation in implicit perceptual-motor learning used in our study and finger tapping tasks which rely on explicitly known motor sequences. Thus an issue of further interest is whether performance improves likewise in dual task conditions by post-training sleep when the rule underlying the sequence of the finger movements is explicitly known to the subject. Based on the present results, sleep-dependent improvements in motor sequence skills, trained under distracting conditions might be even superior for sequences known explicitly than for purely implicitly trained sequences.

## Materials and Methods

### Subjects

A total of 48 students volunteered into the experiment. All subjects underwent a survey to assess whether they met inclusion criteria. The inclusion criteria were the absence of a history of a neurological or psychiatric disease, sleep disorder, un-regular sleep-awake cycle during 6 weeks prior the experiment, intake of medicines and illegal drugs. The study was approved by the local ethic committee, and all subjects provided written informed consent. The subjective activity level was assessed by the EWL-K test [35]: low activity level score (≤3) or high inactivity level score (≥10) were taken as exclusion criterion. Further on, the achievement of more than 50% correct responses on the generation task performed at the very end of the experiment was also used as exclusion criterion, to control for possible explicit knowledge about the procedural tasks pattern. This criterion was chosen because any explicit knowledge about the SRTTs’ sequence is known to bias procedural SRTT performance [20], [36].

Eleven subjects were excluded, based on these criteria. Data of the remaining 37 participants entered the final analysis (sleep group-SG: n = 10, 6 female, mean age = 26.1 years ±3.1 [standard deviation]; nighttime-awake group-NA: n = 11, 8 female, age = 26.0 years ±3.2, daytime-awake group-DA: n = 8, 7 female, age = 28.2 years ±6.3; daytime-awake-subsequent-WPAT group-DAs: n = 8, 5 female, age = 25.9 years ±1.8). The subjects had a regular sleep–awake cycle with an average sleep time of about 7.1 hours ±1.0 [standard deviation] during the 6 weeks prior to the experiment.

### Task

#### Dual-task paradigm

We used a dual-task paradigm for our experiment. In this, two different tasks – a SRTT and a word-pair association task (WPAT) - had to be performed by the subjects in parallel. Each task was embedded in a different modality in order to diminish attentional shifts between tasks. The WPAT was introduced to the subjects as the *relevant task* and accuracy was monetarily rewarded. The SRTT was introduced to the subjects as a *distractor task*. This task delivered the dependent variables for our analysis (reaction times). The hidden structure of the SRTT was not conveyed to the subjects. To prevent slow reaction times on the SRTT, the presentation of word-pairs stopped whenever responses slowed to values >800 msec, and started again after reaction times ≤800 msec were resumed. Thus, slow SRTT performance delayed word-pair presentation and, consequently, lowered the chance of receiving reward.

#### Serial Reaction Time Task (SRTT)

The classical SRTT provided the main task. We used four horizontally arranged squares presented on a computer screen as the visual cue. In each single trial one of the four squares was colored and served as cue to react with a corresponding key press. Subjects rested the four fingers of their non-dominant hand on four response keys (i.e.: v, b, n, and m) of a standard computer keyboard with a sampling frequency of 100 Hz. The spatial configuration of the four response keys was fully compatible with the positions of the squares on the screen. As soon as the required key was pressed, the next cue appeared in a new position, while the preceding cue disappeared. Cues were presented until the correct key was pressed. Reaction times were collected with reference to the cue onset. If the reaction time was longer than 800 msec, a text message demanding faster responses occurred on the screen – this was to prevent the subject from privileging the processing of the paralleled rewarded word-pair association task. The position of the cue within each single trial was given following a *fixed* 12-elements sequence (i.e.: positions 2, 3, 1, 4, 3, 2, 4, 1, 3, 4, 2, 1) or following a *randomized* sequence.

Two experimental sessions were implemented: a first *training session* and a second *retrieval session*.

In the training session, a total of 7 blocks of randomized and fixed sequences of trials alternated, starting from a randomized block. Each randomized block comprised 48 trials whereas each fixed sequence block comprised 180 trials (i.e., 15 repetitions of the fixed 12-elements sequence). However, the fourth, fixed sequence block held 300 trials (i.e. 25 repetitions of the fixed 12-elements sequence) to allow subjects to train the sequence.

The training session began with a practice block consisting in a fixed sequence of 18 trials which were not included in the analysis (Figure 1– left part).

The retrieval session was held after a retention interval which depended on the group assignment. The retrieval session began with a block of 48 randomized trials, which was followed by a block of 180 fixed sequence trials followed again by a block of 48 randomized trials (Figure 1– right part). After the end of the SRTT within the retrieval session, subjects had to perform a *generation task* to analyze the degree of their, eventually acquired, explicit knowledge. The generation task was similar to the SRTT; however, subjects were instructed to no longer react on the current stimulus location but to press the key corresponding to the *next guessed* position of the stimulus. On any of three possible responses that did not correspond to the current stimulus position, the next stimulus in the previously learned sequence became black. If the key corresponding to the *currently* filled square was pressed, the stimulus remained unchanged to prevent the subjects to react in the same way as in the SRTT. The accuracy of the response was not reported to the subject. They had to react on 100 consecutively presented trials, with no marking of the beginning or the end of the repeatedly presented fixed sequence. As for the SRTT, subjects were not told that there would be a pattern of trial repetition.

The distractor task (WPAT) was continuously performed throughout the entire SRTT session. Analysis of performance during training was based on the difference in mean reaction times between the first (marked as “training 1”) and the last (“training 2”) fixed-sequence block and the following random-sequence block, respectively.

#### Word-pair learning

The WPAT consisted of 20 acoustically presented word-pairs taken from the list of the most frequently used German words [37], in order to assume their high familiarity to the participants. The words in each pair were also semantically unrelated (e.g. “leakage” – “fun”). The pairings were unknown to the subjects prior to the experiment. The presentation of each word-pair took 4 seconds (2 seconds for each word). The presentation of word-pairs throughout the dual-task was separated by an interval of at least 4 seconds. The interval became longer whenever SRTT reaction times went above 800 msec. Then the presentation of word-pair was halted until faster reaction times were reached again. The word-pairs had been recorded by a professional speaker and were played in randomized order.

During the training session the SRTT and word-pair association tasks were presented in parallel and were interrupted every 30 seconds. During interruptions subjects were instructed to perform a cued recall of the previously presented word–pairs. Indeed, the first (cue) word of a (pseudo randomly chosen) word-pair was acoustically presented and the subject had to indicate the first letter of the associated word with a mouse-click on the respective character from a list being visualized on the screen. The interruption of the SRTT forced the participants to concentrate entirely on the recall of the word-pairs. For each correct response, the subject gained 50 Euro-Cents on her or his account. The subject got immediate by a counter permanently visible on the screen, being up-dated at each correct response of the so far earned money. After 10 seconds the next randomly selected word of learned word pairs was presented, regardless of whether a response had occurred or not. On every recall block, three word-pairs were cued. Then the dual-task of word-pair and SRTT continued until the end of the SRTT schedule.

#### Daytime awake group without concurrent WPAT

Beside the three groups with a dual-task setting, a further control group was tested, in which the SRTT was performed without concurrent WPAT (i.e., DAs group). Indeed, the SRTT had to be processed alone, without the acoustically presented word pairs and the tasks interruptions for the testing of the learned word pairs. Subsequent to the end of all SRTT blocks on the respective sessions, the 20 word pairs were randomly presented to the subjects every 4 seconds. This presentation was interrupted every 30 seconds for a cued recall of three randomly selected word pairs. Collectively 45 word pairs, chosen by random from the 20 word-pairs of the other groups, were administered by the program. This number of rehearsals reflected the mean number of tests reached by the other groups during their dual-task performance. Beside these changes in the design, all of its other properties matched the test design of the other groups.

### Design and Procedure

Subjects were randomly assigned to four different groups: (i) the *sleep group (SG)*, (ii) the *nighttime-awake (NA)* group, (iii) the *daytime-awake (DA)* group and (iv) the *daytime-awake-subsequent-WPAT (DAs) group*. The groups differed by the length and the timing of the retention interval between the training and the retrieval sessions.

For the sleep group, the training phase took place at 8∶00 PM on the first day. Subjects were instructed to go to sleep at their usual time and to have at least 7 hours of night sleep. After one day without any experimental session, the subjects spent the second night in the sleep laboratory of the Department of Neurology at Lübeck from 11∶00 PM until 8∶00 AM of the following day. Sleep was recorded with standard clinical Polysomnography (PSG) to control for the amount of sleep in each subject. Retrieval session was run in the morning (8∶00 AM) after this second night (see Fig. 1).

The *NA* group had the same procedure, except that subjects stayed awake the first post-training night. An “Actiwatch” (Cambridge Neurotechnology, Ltd.; Cambridge, UK) ensured that subjects did not go to sleep during the entire night. Subjects were free to read books or to chat with fellow study participants in an assigned room in the Department of Neurology at Lübeck (see Fig. 1).

The *DA* group was trained at 8∶00 AM and run the retrieval session at 8∶00 PM of the same day, after a 12-hour daytime period without sleep. The interval was filled with their individual typical student daily-life activities (see Fig. 1). Participants were instructed not to take naps during the day.

We selected groups with differences in circadian phase position for both sessions to allow the comparison between the very different phases of activity and sleep/no-sleep. Therefore differences between the groups should also reflect these differences in the circadian phase position and also the differences between the common daily activities (DA), normal sleep (SG) and loss of sleep for a certain amount of time after learning (NA). Further on, we allowed sleep for a second night for both the sleep and the nighttime awake groups to prevent high reaction times on the retrieval sessions due to fatigue. Further on, the experiments of Stickgold and colleagues [38] let assume a carryover effect of a failed consolidation process that cannot be absorbed by an additional night of sleep.

Finally, the *DAs* group was implemented to test the effects of learning on the sole SRTT. This was a daytime group resembling the design of the daytime-awake group, but performing no concurrent word-pair distractor task.

For each subject the experiment began with the assessment of handedness, general sleep quality and subjective activity by the Edinburgh Handedness Inventory, (EHI) [39], the Pittsburgh Sleep Quality Index (PSQI) [40], and the “Eigenschaftswörterliste” (EWL-K) [35], respectively. Subjects were asked for prior intake of medicines, illegal drugs, caffeine, and alcohol, as well as present or prior diseases or disorders. Working on night shifts within six weeks prior to participation was further enquired. These tests and the questioning lasted for about 10 to 15 minutes for each subject. The subsequent training session lasted 15 to 20 minutes according to the subject’s individual speed. After the training, subjects were dismissed for sleeping; sleep deprivation or their daily activities, according to their group assignment.

The *SG* group and the *NA* group were allowed to sleep the second night after the training session. In the morning after the second night, that is, at the *retrieval session*, we assessed the sleep duration and the subjective quality of sleep for both the groups. Additionally, the subjects of the *SG* group were asked about the duration of the first night sleep after training: the sleep quality was self-assessed by a questionnaire. All four groups performed again the EWL-K prior to the start of the retrieval task in order to assess the subjects’ activity level. These tests and surveys lasted for about 10 minutes for each subject. The subsequent retrieval and generation tasks also lasted for about 10 minutes. The monetary reward was paid to the subjects directly after the end of the session.

### Data Analysis

Reaction times (RTs) were collected in the SRTT. We sought to investigate the changes in performance across the training session and from the training to the retrieval sessions. On the one hand, SRTT performance during the training session was measured by subtracting the RT performance at the first and the last fixed sequence blocks (block 2 and block 6, respectively) from the RT performance to their subsequent, random sequence blocks (block 3 and block 7). The two measures being obtained were referred to as *Delta Training 1* and *Delta Training 2* (Figure 1, left part).

On the other hand, SRTT performance at the retrieval session was calculated by subtracting the RT performance at the fixed sequence block (block 9) from the RT performance to its subsequent, random sequence block (block 10). The measure being obtained was referred to as *Delta Retrieval* (Figure 1, right part).

A first *Group* (SG vs. NA vs. DA vs. DAs) × *Time-points* (Delta Training 1 vs. Delta Training 2 vs. Delta Retrieval) analysis of variances (ANOVA) was performed to investigate SRTT performance across all time-points of assessments.

Then, two separate ANOVAs were run. The change in performance across the training session in the four groups was investigated through an ANOVA including *Group* and *Training* (Delta Training 1 vs. Delta Training 2) as between- and within-subjects factor, respectively. The change in performance from the training to the retrieval session, indeed the presence of off-line gains in procedural skill, was investigated through a similar ANOVA with *Group* and *Retrieval* (*Delta Training 2* vs. *Delta Retrieval*) as between- and within-subjects factor.

A *p*-value <.05 was considered significant. Two-sided post-hoc Scheffé tests and paired-sample *t*-tests were used to compare performances between and within groups, respectively.
